# “You Don’t Get That from Professionals”: A Consumer-Led Peer Recovery Program for Families and Friends of Individuals with Alcohol and Other Drugs Use Issues in Darwin

**DOI:** 10.3390/ijerph20085514

**Published:** 2023-04-14

**Authors:** Noemi Tari-Keresztes, Noelene Armstrong, James A. Smith, Himanshu Gupta, Sam Goding, Sal-Amanda Endemann

**Affiliations:** 1 Rural and Remote Health, College of Medicine & Public Health, Flinders University, Casuarina, NT 0815, Australia; 2 Northern Territory Lived Experience Network, Darwin, NT 0820, Australia

**Keywords:** alcohol and other drugs, empowerment, families and friends, peer support, personal recovery

## Abstract

While there has been a reduction in alcohol consumption among Australians aged 18 years and above, about 25% of people still drink above the recommended limit. The use of alcohol and other drugs is a substantial issue in the Northern Territory; however, there have been significant investments in alcohol reforms over the past few years. This paper reports on a pilot study that involved co-designing, implementing, and evaluating the Circles of Support consumer-led recovery and empowerment program for families and friends of individuals with alcohol and other drugs use issues. The evaluation comprised a mixed-methods approach; however, this article only presents the qualitative component (*n* = 7). Interview data were thematically analysed, and four main themes were identified: (1) the value of a peer-to-peer approach; (2) facing challenges and distress; (3) adopting self-care strategies; and (4) the development of valuable skills. Participants enjoyed the program content and learning. This involved self-care and communication strategies, boundary setting, service navigation, the concept of post-traumatic growth, the circles of control, and the stages of change model for families. Our findings strongly support the scaling up of the program in Darwin and other locations across the Northern Territory and future program adaptation for different vulnerable target audiences.

## 1. Introduction

In this paper, we introduce the Circles of Support program (hereafter program) and present evaluation findings from the recent ‘Supporting family members’ and friends’ individual recovery with a locally co-designed peer-led recovery program in Darwin’ pilot project. This program is a consumer-led recovery and empowerment program for families and friends (hereafter families) of individuals with alcohol and other drugs use (hereafter substance use), with the issues being co-designed by the Northern Territory Lived Experience Network (hereafter Network), which involves persons with lived experiences of substance use and related challenges. The program development and evaluation were funded through the Alcohol and Drug Foundation, Information Support Services Family and Friends Grants Program, from funding provided by the Australian Government Department of Health.

The program is delivered by skilled peer facilitators affiliated with the Network who followed a stepped vocational pathway that was found to be the ideal pathway for the emerging local peer workforce [1]. Participants in the program learn about the following topics: (1) mental health and alcohol and drug-related misuse and co-occurring issues; (2) recovery and ways to support recovery; (3) identifying and responding to a crisis; (4) ways to support own wellbeing and practice self-care; (5) managing overwhelming emotions and responses; (6) setting boundaries on relationships; (7) effectively communicating their needs and rights; (8) responding to stigma and discrimination; and (9) navigating the mental health, alcohol, and other drugs service system. They also participate in regular co-reflection sessions where they are able to provide feedback to peer facilitators, which offers an opportunity for continuous quality improvement. [2].

### 1.1. The Landscape of Substance Use

According to the Australian National Health and Medical Research Council guidelines, healthy men and women should drink no more than ten standard drinks a week and no more than four standard drinks on any one day [3]. While there has been a reduction in alcohol consumption among Australians aged 18 years and above, about 25% of people still drink above the recommended limit [4]. Data published in 2019 also showed concerns about illicit drug use among Australian adults. For instance, a significant increase was seen in cannabis, hallucinogen, ecstasy, inhalants, ketamine, and cocaine use [5]. While there have been significant investments in alcohol reforms in the Northern Territory (hereafter the Territory) over the past few years [6], substance use is a substantial issue. According to a recent report, the estimated per capita alcohol consumption in the Territory for persons aged 15 or over in 2017 was 11.6 litres per person [7]. The social and economic costs of alcohol consumption are estimated to be $1.38 billion per year [8]. In addition, mental health, suicide, and substance use disorders make up about 36% of the total burden of disease, which is three times the national average [9].

### 1.2. The Harm Caused by Substance Use

Substance use issues rarely impact the individual only; they also significantly affect the whole family and even the broader social environment [2,10]. For instance, it may strain relationships, create social isolation, and weaken families’ resilience as well as their physical, emotional, and financial security [11]. In addition, family members’ studying, employment, quality of life, and physical and mental wellbeing are also often negatively impacted [12,13].

Families of individuals with substance use issues often fulfil unique carer roles [14]. They are more vulnerable than others who care for individuals with other challenges, such as mental health, disability, and chronic conditions [15]. Drug use is one of the most stigmatised behaviours and is often perceived as a weakness or an undesirable behaviour that should be controlled [16]. Hence, the families of these persons are also often blamed by others and seen as likely to be ‘contaminated’ by the family member’s drug use [16,17]. More commonly, caregivers can face stigmatisation that involves secondary [18], courtesy [19], associative [20], or affiliated stigma [21]. These all refer to the public stigma felt by families, which manifests as a sense of shame and inferiority [17]. Hence, stigma awareness education should include messages such as ‘recovery is possible’ and ‘no one is to blame’ [22].

### 1.3. Stigma and Challenges Experienced by Families

Many families of persons with substance use issues withhold information from their social environment to avoid stigma and negative judgements [16]. Indeed, past studies have described that concealing this information negatively impacts self-esteem, life satisfaction, happiness, depression, and anxiety because keeping a secret of this nature can be psychologically demanding [23]. Additionally, families often withdraw from social interactions to avoid judgement [2,12]. These avoidance behaviours can significantly burden the families’ quality of life, leading to mental health challenges, the experience of chronic loneliness, maladaptive coping strategies, and even substance use issues [15,24]. Thus, it is necessary to pay attention to families’ mental wellbeing and to support them in their own recovery journey to enable them to walk alongside their loved ones who are experiencing substance use issues [2,25,26].

Previous studies have shown that help-seeking among families is relatively low [27]. They are often reluctant to seek help for themselves because of stigma, shame, and a lack of information [2,16,17,27], which may result in neglecting their needs and health [16]. In addition, families often describe that when they accessed services for their loved ones, they were rarely asked about their support needs, challenges, general health, or mental wellbeing [28]. Among the available support for families, services assisting families in education, paid employment, respite, transport, and in-home support are usually available in Australia [28,29]. Peer support is another available form of support that can provide a safe, trusting, non-judgmental, inclusive, and shared space where families feel accepted and understood [2,30]. In Australia, national policies and forums advocate for recovery-oriented practices informed by people with lived experience [31,32]. Numerous peer support groups, forums, and resources are commonplace, including peer support for families by various organisations [33,34,35]. However, in the Territory, peer workers are poorly utilised in delivering psychosocial support activities [36,37,38,39]. Yet, evidence shows that peer support for families has numerous benefits, such as on their wellbeing and individual recovery [2,40,41,42].

## 2. Materials and Methods

### 2.1. Aims and Objectives

The evaluation primarily aimed to co-design, implement, and evaluate a local, consumer-led recovery and empowering program for families. The objectives were to assess the appropriateness and effectiveness of the program in terms of (1) exploring the main stressors and challenges faced by families on their journey of supporting someone with substance use issues; (2) assessing the families’ mental wellbeing; (3) investigating the families’ experiences with the program; and (4) considering the program’s impact on the families’ mental wellbeing. This article will only describe the families’ experiences with the program, which involved reflections on their learnings and the most helpful program elements. Further evaluation findings were published in the project report [2].

### 2.2. Evaluation Design

The suitable evaluation approach was co-designed in collaboration with the evaluation team, the Network, and other lived experience representatives, including the student cohort of the first local Certificate IV in Peer Work course. It applied a mixed-methods approach involving a co-design workshop, individual interviews with families and facilitators, and a survey at the start and end of the program. However, this article only presents the qualitative evaluation component, including data collected from families through in-depth, semi-structured individual interviews.

This study had four stages, namely ‘Development’ (Stage 1); ‘Implementation’ (Stage 2); ‘Evaluation’ (Stage 3); and ‘Review’ (Stage 4). Stage 1 included a narrative literature review to identify relevant evidence-based practices and research tools and a consultation workshop held in April 2022 with local lived experience representatives and stakeholders. The workshop aimed to inform the program content, design, evaluation approach, and research tools used.

The individual, semi-structured, in-depth interviews included questions about the families’ backgrounds, wellbeing, the reason for participating in the program, and the main characteristics and stressors regarding the family/carer role. It assessed their overall experience with the program, modules, and activities and identified the possible areas for improvement and challenges associated with the program.

Ethics approval for the evaluation was obtained from the Northern Territory Department of Health and Menzies School of Health Research Human Research Ethics Committee (HREC 2021-4164).

### 2.3. Sample and Recruitment

The program was offered between April 2022 and September 2022 for families in various locations in Darwin: Winnellie, Casuarina, and Palmerston. Three programs were delivered in small groups. For the sake of the families’ convenience, the program was offered via morning, afternoon, and evening classes, with the latter being the most accessible for them. Each program was facilitated through self-referral and was delivered over nine weeks in a three-hour session. In total, 19 people participated in the program. Each group comprised between five and seven participants. One participant started the program but could not finish it because this person worked in the mental health sector and felt overwhelmed participating in the program after hours.

The evaluation applied the following inclusion criteria: (1) being at least 18 years of age; (2) living in Darwin/Palmerston; (3) being a carer for people with lived experience of substance use issues; and (4) being able to provide informed consent. The sampling method was purposive. The peer facilitators informed families about the evaluation component at the beginning of the program. Interested families were provided with a Participation Information Sheet, which included information about the project, evaluation team, evaluation approach, risks and benefits of partaking in the evaluation, ethics approval, and contact information for further enquiries. Additionally, a Consent Form was handed to families. Participation in the evaluation was voluntary and did not impact participation in the program. The evaluation team followed up with interested families, and those who provided consent to partake in the evaluation were interviewed online (*n* = 7) by an evaluation team member (NTK). This approach was deemed useful for increasing families’ safety and trust and improving accessibility to this vulnerable population [1,37,43]. The interview lasted approximately 45–60 min. Families participating in the evaluation received a $30 grocery voucher (that cannot be used to purchase alcohol, tobacco, or gambling products) to acknowledge their time and contribution to the study.

Table 1 summarises the families’ sociodemographic data. They had similar backgrounds in relation to gender and age as in previous relevant large-scale national studies [15,28,44]. However, our sample had higher education and employment rates [2]. In addition, while the Territory has a diverse population and is considered one of the most disadvantaged regions in Australia [45], most participants in our study were Australian-born, non-Indigenous people and spoke English at home as their first language. Some families cared for more than one person and experienced significant challenges due to their complex carer roles. These adversities involved being a carer for numerous persons with various challenges, difficulties navigating services and getting support, violence, no acknowledgment of their hard work, no respite, and challenges relating to their own lived experience [2].

### 2.4. Data Analysis

All interviews were transcribed verbatim. Families were denoted as program participants (PPs), and a number was allocated to protect their anonymity in the presentation of the findings. The data were managed using NVivo software and analysed thematically. This method systematically identifies, organises, and offers insight into patterns of meaning (themes) across a data set [46]. The data analysis was underpinned by the Grounded Theory principles [47]. This inductive, data-driven, bottom-up approach privileged participants’ voices and lived experiences. Two team members (N.T.-K. and H.G.) individually carried out the initial coding. The discrepancies in the coding were sorted through discussions between the coders, and the resulting themes were then finalised. Four themes were identified relating to participants’ experiences with the program: (1) the value of a peer-to-peer approach; (2) facing challenges and distress; (3) adopting self-care strategies; and (4) the development of valuable skills.

## 3. Results

This section presents the evaluation findings of the interviews conducted with families. This includes their perspectives and experiences relating to the four identified themes listed above.

### 3.1. The Value of A Peer-to-Peer Approach

In the last couple of years, a lot has been achieved regarding utilising peers in psychosocial support programs in the Territory [1,2,36,37,48]. However, available peer education and recovery programs are still scarce in the local community, especially among families of people with substance use and mental health challenges who often prioritise the persons’ needs and neglect their own mental wellbeing. Thus, participating in the program was the first time many participants experienced peer support that focused on their own empowerment and mental wellbeing. Previously, most of them had not engaged with any support for themselves. Instead, they had prioritised the person’s needs they cared for and had neglected their own needs and health.

*“At the start, I thought that I don’t…I can’t have this opportunity [to seek help for myself] because I have to support them …you always have to worry and…your priority is to help them.”*

(PP7)

The families’ primary goals with the program was to connect with others and learn. They wanted to meet people with similar challenges and learn and understand more about the issues they face.

*“[I decided to] sign up to be a member of [the program] just to sort of keep in touch with what goes on in the world in regard to supporting people with lots of issues. My expectation was to learn and understand more about the issues…”*

(PP5)

They were amazed by the power of the peer approach that created a safe, non-judgmental, and trusted environment where participants felt included, valued, eased, and understood.

*“The peer connection and support [were] the most important. You felt safe. You felt that you were not alone…you knew that you could trust them… no judgmental and all that…it was the best thing that I [could] do for myself.”*

(PP7)

Families enjoyed the facilitation and liked the content, which exceeded their expectations. The facilitators introduced regular breaks with snacks and implemented various activities. This included open discussions, reflection, crafts, and activities for individuals and pairs. They also created the physical space in a purposeful way to be comfortable for everyone. For instance, many sensory objects were available to those needing them.

*“100% satisf[ied], the contents being fantastic. You know, [it] touched on some really, really good themes…I can learn from peer people with shared experience and from conversations with people who have similar backgrounds and are willing to be vulnerable and open about their backgrounds and share what they know from a deep place of understanding, [which] is really valuable. You don’t get that from professionals.”*

(PP1)

Families are highly stigmatised by alcohol and drug use. Thus, the perception of stigma and shame that prevent people from asking for help and participating in peer support programs, especially in small places like Darwin, needs to be considered.

*“Darwin’s a small place; you may know people that come along…day one we had people coming in…[and] there was a [person] that I used to work with… [this person] got up and went outside… I guess it is the stigma that [this person] might feel and also that confidentiality… [this person] had an issue with trusting… it was just unfortunate… shame or stigma or trust.”*

(PP5)

### 3.2. Facing Challenges and Distress

Support programs aiming to empower families and improve their skills to maintain good mental health are critical since families face significant challenges while they support and care for their loved ones. As the participants expressed, they can get distressed, exhausted, isolated, and have negative feelings and experiences.

*“I felt incredibly isolated… and I didn’t really know where to turn for help…. I did not know what the future possibly could look like… And I think just probably like it an overall feeling of just being overwhelmed.”*

(PP4)

Families shared that stigma, shame, and guilt were some of the most critical challenges they faced, which led them to have additional distress, withdraw from social interactions, and ask for help. This included informal support from the broader family and other friends. The situation was even worse for people with culturally and linguistically diverse backgrounds since cultural beliefs, norms, and values often exacerbated stigma.

*“…trying to explain it to family…this sort of shame and guilt and all the things that come around it… I just found it really difficult to talk to [them] about the situation… that was quite sort of distressing as well”*

(PP4)

Additionally, some families developed confidence in this role and navigating the system over the years; however, some felt lost, unsupported, and doubtful. Many families also described various indicators and symptoms of stress. Among them, gagging, anxiety, and panic attacks were often mentioned. Sleeping problems, unhealthy eating, and inappropriate reactions to situations, such as being snappy and sharp when communicating with others, were also associated with distress.

*“In the beginning, I wasn’t confident I was actually scared.”*

(PP6)

They agreed that self-awareness and breaks to maintain their own mental wellbeing and support their loved ones were critical. However, this was not always a possibility for some of them.

*“I try to [have a break], then some days there are no breaks because you have to [be] there as a carer.”*

(PP1)

### 3.3. Adopting Self-Care Strategies

Not everyone had a solid self-care strategy at the program start; however, some families had already implemented self-care strategies before they joined the program. This often involved physical activity and other healthy lifestyle elements such as healthy eating, proper sleeping, and not consuming alcohol. In addition, reaching out for professional help if necessary and dedicating time for themselves were also mentioned. Others emphasised quieter activities that helped them calm down, such as a massage, relaxation, music, hot showers, and easy walking in nature. Many families mentioned friends and other social groups as significant parts of their self-care strategies.

*“No concept of [self-care] at all. And in fact, in the [Circles of Support] classes, we joke about how I’m learning about self-care now… Yeah, it was just the way life was. It was the way I was brought up. It’s the way things were. You just got on with it… I’ve learned that there are minor forms of self-care…That I’ve been practising, but nothing like I heard in the group from participants and the facilitators.”*

(PP3)

Seeking help from professionals was also considered a critical self-care strategy which required self-reflection skills and knowledge about how and where to ask for help.

*“I’m engaging with…[an] employee assistance program… So, I reach out, I know [how] to reach out….”*

(PP2)

### 3.4. Development of Valuable Skills

They shared the most valuable skills and knowledge they learnt. One was the concept of post-traumatic growth [49], which was new for most participants. This meant becoming empowered through understanding the possible positive growth following the adversity they had experienced.

*“The post-traumatic growth, which is something we all learned about in the Circle [of Support program], and everybody latched onto it. Wow, that’s fantastic. [one participant] was the only one who’s heard of it before because [they have] done classes with [the lived experience network]… And it’s something I knew intuitively and that it gives me the resilience that I have because I can keep taking the knocks and that they might knock me down for a day or two. However, then I’m back at it.”*

(PP3)

Families also shared that the stages of change for families model [50], which is not widely used in family support services in the Territory, helped them understand their journey and learn how to cope with their situation, depending on their stage.

*“Stages of change [for families]… as soon as I saw [it]..that is our family. That’s exactly…sort of what way we’re going through so…[it] was kind of revolutionary to me because it just suddenly very clearly mapped out the journey that we’ve been on, and I guess it’s comforting to know because it all just feels so chaotic when you’re in the middle of it. It was kind of comforting to know; Oh, there’s a psychology behind this as well. And that’s what we’ve all been through…”*

(PP4)

Families highly rewarded the circles of control [51] model and its visual presentation since it reminded them that some things were out of their control. Instead of getting stressed and upset about these things, they learnt that focusing on what they could change would improve their stress management skills.

*“Articulating that what’s within our control and outside of our control, which is about our thinking, was certainly a fundamental piece for me—and reinforcing that about the ways to think about the person who is mentally ill or dependent on alcohol. A lot of what they’re doing is entirely out of our control. And therefore, where we can focus our energy and attention on what aligns with our values…”*

(PP1)

As it was previously described, self-care was a significant part of the program that participants appreciated. The peer environment allowed participants to share and discuss their own strategies in a safe and trusted environment. They also learnt from each other, and many activities targeted improving these skills.

*“I get stuck on [with self-care activities]… what that looks like, and you think I just have a warm shower or go for a walk and just forgetting that… [however] there’s a lot of other things that you can do to nurture yourself so that looking at that list [we received in the program] and picking some of those things out and seeing what works on a daily basis…one size doesn’t fit all, so it’s just working out what will work for me.”*

(PP5)

Communication with a person with substance use issues is often challenging. The program got participants to understand how inappropriate communication strategies could contribute to escalating heated and unpleasant situations, such as talking about the impact of substance use issues or trying to convince the person to seek help. However, learning about communication styles that help families to de-escalate these situations and better support the conversation with the person was powerful. Families described that using “I” statements, avoiding blame-focused discussions, and finding the right environment and time to address these issues were vital.

*“I have a really troubled way of communicating with one of my [children], and we seem to buttheads no matter what we do, it always ends in an argument… So I think, you know, really looking at that communication with [them] and how I could change that… stopping and reflecting and having a good look at myself and how I communicate, not just putting it all on [them] and [their] addiction, but you know how I possibly become part of the problem when trying to have this communication.”*

(PP5)

Improving families’ boundary-setting skills was also a significant part of the program, with many participants having struggled with it previously. However, by the end of the program, they had learnt that they needed to practise these skills regularly and communicate these boundaries to maintain their own mental wellbeing, prevent exhaustion and emotional and financial burnout, and support their loved ones appropriately.

*“……if you are a rescuer, then…you blur the boundaries because you wanna fix…you wanna help, but actually you’re making it worse because you’re increasing the dependency. So, the work in boundary setting and communicating the boundaries [are very important] so that you get what you need out of life.. Every day, they need to be set and set and set.”*

(PP1)

The families were given a comprehensive list, including local services and their contact details, which can help them to navigate the support system. They also appreciated the skills they learnt to respond to a crisis and advocate for their loved ones. After the program, they felt empowered and confident.

*“If times get tough again with my [child] that, I will know how to reach out…. Particularly to the lived experience network. I think that’s really that was a key learning I think… finding out that some people can support navigate the system…[and] understanding about rights and responsibilities and things like that within the system that we, we have to negotiate… I’ve felt a bit more empowered in that, and so when we were going through the [service] stuff recently, it was just incredibly, you know, people can be very patronising to [my child] and ignore [my child’s] right…. Advocating for [my child] and making sure that is [treated], you know, with respect.”*

(PP4)

Exploring values and making families recognise how important it is to align them with their behaviour was another critical point of the program. Developing new self-care strategies based on families’ value systems and preferences was a new concept for many.

*“[the facilitators] worked hard on the values piece…and aligning our behaviours, oursel[ves], gratitude with our values…once you know that your values are aligned…[your] thinking blinds up. You experience that tension when you’re going away from values, so they worked hard with that, creating the boundaries.”*

(PP1)

## 4. Discussion

This study addressed a significant gap in the Territory’s service provision and evidence base by developing, implementing, and evaluating a local consumer-led recovery and empowerment program for families of persons with substance use issues. It also highlighted families’ reflections on their learnings, including the most valuable skills they had acquired to support their own mental wellbeing and recovery.

Peer support groups, forums, and resources, including peer support for families, are commonplace in other jurisdictions of Australia [33,34,35]. However, for most families in Darwin, this program was their first experience with peer support, noting that in the Territory, psychosocial support activities delivered by peers are seldom available, especially for families of people with mental health and substance use issues, which are very sparse indeed [37]. It also reflects on the evidence that families often prioritise individuals’ needs and neglect their own [2,16,17], as many shared in this study. As previous studies showed [2,11,12], most families felt isolated, so their main motivations were to connect and learn. The peer-only environment provided a safe space where families felt understood and valued, which allowed them to grow, be empowered, and improve their own mental health [41,42]. However, perceived stigma, commonly experienced by families of persons with substance use issues [12,15,16,23,24], still prevented some of them from joining the program.

Similar to previous studies [15,52,53,54], individual interviews highlighted how challenging it is for families to walk alongside and support the journey of their loved ones. They experienced high levels of distress with various physical, psychological, and behavioural symptoms, isolation, and negative feelings being experienced on their own journeys. Some of them also felt lost and unsupported. While families thought that respite should have been a priority, sometimes this was not possible for them.

Some families had already practised self-care; however, some did not have solid strategies before the program participation. Since self-care was a significant part of the program, they learnt new techniques from each other and attempted to put them into practice. They enjoyed exploring their own value preferences and aligning their self-care activities with them, which may provide a long-term positive attitude when it comes to continuing practising them.

Among the most valuable learnings and skills, families mentioned the concept of post-traumatic growth [49], the stages of change for families model [50], the circles of control model [51], self-care and communication strategies, boundary setting, and comprehensive information about service navigation. These helped them identify the stage of their own situation, learn how to cope with challenges, reduce stress, develop hope, experience growth, create a better and more supportive relationship with their loved ones, and implement self-care on a regular basis [2].

### Study Limitations

While the pilot project was well-received and deemed successful in learning valuable skills and supporting families in recovery and empowerment [2], it had some limitations. While a small sample size and purposive sampling are appropriate in a qualitative study, this pilot project accessed nineteen participants, and only seven participated in the qualitative evaluation. This might be related to the project time and budget constraints. However, it provided critical data about the need for peer support programs for families with unmet needs in the Territory and helped build the evidence base of the program.

## 5. Conclusions

Families appreciated the availability of this local consumer-led course and enjoyed the skills they learnt. They liked the purposefully built program content. This included various concepts that families highly valued. This pilot project described a high demand for peer support among families in the Territory, which reflects the lack of available support for this vulnerable population in this jurisdiction. It also drew attention to further work that needs to be done to raise awareness about peer support and reduce the shame and stigma experienced by families in the local community. Our findings support the idea of scaling up the program in Darwin and other locations across the Territory and aid future program adaptation for different caregiver settings.

## Figures and Tables

**Table 1:** Sociodemographic profile of families (program participants).

Variable	Frequency (%)
Gender	
Male	25.0
Female	68.75
Other	6.25
Age Category	
18–24 yrs	6.3
25–34 yrs	6.3
35–44 yrs	25.0
45–54 yrs	31.3
55–64 yrs	18.8
65+ yrs	12.5
Country of birth	
Australia	75.0
Other	25.0
The main language spoken at home	
English	100.0
Other than English	0.0
Aboriginal and Torres Strait Islander	
No	87.5
Aboriginal and Torres Strait Islander	12.5
Highest level of education	
Less than Year 12	0.0
Year 12 or equivalent	18.8
Vocational education	25.0
Diploma course	6.3
Bachelor’s degree	37.5
Postgraduate degree	12.5
Employment	
Employed	62.5
Self-employed	12.5
Student	6.3
Unemployed	0.0
Unable to work	0.0
Retired	6.3
Other	12.5
Relationship status	
Single	43.8
Relationship	0.0
Married/de facto	50.0
Other	6.3
Number of children	
0	25.0
1	12.5
2	31.3
3	18.8
4	12.5
Relationship with the person they care for	
Partner	25.0
Parent	31.3
Child	12.5
Sibling	25.0
Other	6.3
Caring for more than one person	
Yes	25.0
No	75.0

## Data Availability

Data not available on request due to privacy/ethical restrictions.
